# The Evolving Role of Cannabidiol-Rich Cannabis in People with Autism Spectrum Disorder: A Systematic Review

**DOI:** 10.3390/ijms252212453

**Published:** 2024-11-20

**Authors:** Bilal Jawed, Jessica Elisabetta Esposito, Riccardo Pulcini, Syed Khuram Zakir, Matteo Botteghi, Francesco Gaudio, Daniele Savio, Caterina Martinotti, Stefano Martinotti, Elena Toniato

**Affiliations:** 1 Unit of Clinical Pathology and Microbiology, Miulli Generale Hospital, LUM University, 70021 Acquaviva delle Fonti, Italy; bilaljawed2007@gmail.com (B.J.); khuramabbas512@gmail.com (S.K.Z.); martinotti@lum.it (S.M.); 2 PhD Program in Science and Technology in Sustainable Development, G.d’Annunzio University, 66100 Chieti, Italy; riccardo.pulcini@unich.it (R.P.); caterina.martinotti@hotmail.it (C.M.); 3 Department of Innovative Technology in Medicine and Dentistry, G.d’Annunzio University, 66100 Chieti, Italy; j.elisabetta.esposito@gmail.com; 4 PhD Program in Innovative Technologies in Clinical Medicine and Dentistry, G.d’Annunzio University, 66100 Chieti, Italy; 5 Department of Clinical and Molecular Sciences, Università Politecnica delle Marche, 60121 Ancona, Italy; matteo@botteghi.com; 6 Unit of Hematology, Miulli Generale Hospital, LUM University, 70021 Acquaviva delle Fonti, Italy; gaudio@lum.it; 7 Research & Development Unit, R&D Solution Srl, 13030 Greggio, Italy; info@rdsolutions.it

**Keywords:** cannabidiol, CBD, autism, autism spectrum disorder

## Abstract

Autism spectrum disorder (ASD) is a neurological disease and lifelong condition. The treatment gap in ASD has led to growing interest in alternative therapies, particularly in phytocannabinoids, which are naturally present in *Cannabis sativa*. Studies indicate that treatment with cannabidiol (CBD)-rich cannabis may possess the potential to improve fundamental ASD symptoms as well as comorbid symptoms. This systematic review aims to assess the safety and efficacy of CBD-rich cannabis in alleviating the symptoms of ASD in both children and adults, addressing the treatment gap and growing interest in CBD as an alternative treatment. A comprehensive literature search was conducted in February 2024 using the PUBMED and Scopus databases while following the Preferred Reporting Items for Systematic Reviews and Meta-Analyses (PRISMA) guidelines. The search focused on studies from 2020 onward involving human populations diagnosed with ASD and treated with CBD. Four studies met the inclusion criteria and were analyzed. The review included 353 participants with ASD from studies conducted in Israel, Turkey, and Brazil. The studies varied in design, sample size, dose, and treatment duration. Dosages of CBD were often combined with trace amounts of THC. Improvements were noted in behavioral symptoms, social responsiveness, and communication, but cognitive benefits were less consistent. Adverse effects ranged in severity. Mild effects such as somnolence and decreased appetite were common, while more concerning effects, including increased aggression, led to some cases of treatment discontinuation. CBD-rich cannabis shows promise in improving behavioral symptoms associated with ASD. However, variations in study designs, dosages, and outcome measures highlight the need for standardized assessment tools and further research to understand pharmacological interactions and optimize treatment protocols. Despite the mild adverse effects observed, larger, well-controlled trials are necessary to establish comprehensive safety and efficacy profiles.

## 1. Introduction

Autism spectrum disorder (ASD) is a neurological disease condition characterized by difficulties in communication and social interaction and the manifestation of repetitive behaviors. The prevalence of the symptoms varies among individuals, ranges from mild to severe, and frequently impacts their daily functioning and overall quality of life [1]. It is a lifelong chronic condition which develops through a series of biological processes or environmental factors, such as inflammation or oxidative stress. This ultimately leads to a pleiotropic metabolic impact, resulting in a wide range of symptoms of ASD [2,3].

Approximately 1 in 100 children globally is thought to have autism [4]. This estimate is an average figure, and the reported prevalence differs significantly among studies. Some well-controlled studies have, however, documented figures that are substantially higher. In Italy, the prevalence of ASD is rather high, with approximately 1 in 77 children between the ages of 7 and 9 years being diagnosed with autism. Males are 4.4 times more likely than females to have ASD [5].

ASD not only affects the individuals diagnosed but also has a significant impact on their families, caregivers, and communities. Tailored assistance and treatments are necessary to effectively meet the distinct requirements and challenges faced by individuals diagnosed with ASD. This condition is diagnosed after a thorough evaluation and assessment by a qualified clinician and is primarily treated through symptomatic treatment and educational and behavioral services [6,7]. Although significant research has been conducted, there is still no medicine approved by the Food and Drug Administration (FDA) which precisely targets the fundamental symptoms of ASD [8].

The treatment gap in ASD has led to growing interest in alternative therapies, particularly those with phytocannabinoids, which are naturally present in *Cannabis sativa*. Plant extract from *C. sativa* has been used medicinally since ancient times (approximately 2900 BC in China and 1000 BC in India), mainly for analgesic, anti-inflammatory, and hypnotic purposes [9]. The endocannabinoid system (ECS) modulates numerous bodily functions either in sickness or in health, which include exercise, appetite, sleep, inflammatory pain, mood, and memory. Endocannabinoids (eCBs) and their receptors are found in the neurological system, glands, connective tissue of internal organs, and immune system, and these eCBs are synthesized on demand and modulated by ligands [10]. The ECS is a neuro-modulatory system composed of functional endogenous eCBs, mainly anandamide (AEA) and 2-arachidonoylglycerol (2-AG) [11,12], and receptors, including cannabinoid type 1 and type 2 receptors [13]. CB1 and CB2 are involved in emotional regulation and social responsiveness [14]. The underlying pathophysiologies of ASD are thought to underlie immune dysfunction, aberrant synaptic plasticity, and metabolic disturbances, and the ECS can have a modulatory role in these mechanisms [15]. Individuals with ASD often show immune dysregulation, leading to neuroinflammation and elevated cytokines such as IL-1β, TNF-α, and IL-6 [16,17]. This disrupts brain function and synaptic plasticity and contributes to social and communication challenges. ECS regulates synaptic plasticity and neurotransmission. Overactive microglia exacerbate synaptic impairments. Lower AEA levels in individuals with autism may impair social behaviors, while the ECS helps modulate neurotransmitters and protect against neuroinflammation [18]. Meanwhile, eCBs regulate cell differentiation and proliferation by activating different pathways. The process also affects the uptake and metabolism of endocannabinoids through certain enzymes like FAAH, which are responsible for the synthesis and breakdown of cannabinoids like 2-AG and AEA [19,20]. The ECS may operate differently in neurodevelopmental disorders, although the specific mechanisms involved have not yet been clearly established [21,22]. Targeting the ECS presents a promising therapeutic strategy for ASD [23,24].

It has been reported that lower concentrations of eCBs, mainly AEA in patients with ASD, have been linked to various symptoms and behavioral challenges associated with autism [25,26]. Additionally, reduced levels of oleoyl ethanolamide and palmitoyl ethanolamide have been observed in ASD patients which are related to AEA [27]. These molecules are involved in key physiological processes such as appetite regulation and neuroprotection by reducing cerebral oxidative stress through binding to PPARγ [28]. The main medically relevant constituents of the cannabis plant (phytocannabinoids) are Δ9-tetrahydrocannabinol (THC), which is a psychoactive component, and cannabidiol (CBD), which has non-psychotic opposing effects and appears to have antiepileptic, anxiolytic, and neuroprotective properties [29,30]. CBD is a highly prevalent extract derived from *C. sativa*, and its uses were more focused in western healthcare systems during the early 19th century [31]. It has been reported to enhance social communication and behavioral regulation and decrease aggressive states of mind [32,33]. Several recent reviews have indicated that treatment with CBD-rich cannabis may possess the potential to improve fundamental ASD symptoms as well as comorbid symptoms [34,35,36]. CBD is an allosteric modulator of CB1 which antagonizes the effects of THC, which is an agonist at CB1 and produces a sense of euphoria. The major psychoactive component of cannabis is THC, which acts on the CB1 receptor in the CNS, and in comparison, CBD does not significantly affect CB1 or CB2 receptors intrinsically to produce its effects. CBD can inhibit THC’s ability to activate them fully, thereby reducing its psychoactive impact [37]. For medicinal benefits without significant euphoric symptoms, the synergistic effects of higher dosages of CBD and low traces of THC are frequently used [38]. CBD may appear to produce its effects through receptors such as glycine α3 and α1, TRPV1, TRPV2, 5-HT1A, GPR55, GABA, and PPARγ [39,40,41]. CBD has been found to raise the serum levels of AEA by inhibiting the enzyme FAAH. Additionally, it increases adenosine availability by blocking its reuptake, which may help explain its anti-inflammatory and neuroprotective effects [42].

Previous reviews primarily examined the impact of medicinal cannabis on different neurological health outcomes. With the increasing interest in CBD, our systematic review seeks to explore its potential benefits for ASD. The scarcity of well-controlled trials before 2020 has left a gap in the evidence regarding the efficacy, safety, and tolerability of CBD-rich cannabis as an alternative ASD treatment. Our systematic review aims to address this gap by summarizing and analyzing the latest evidence from relevant human clinical studies on autism patients only to assess the safety and efficacy of CBD-rich cannabis in alleviating ASD symptoms in both children and adults. We also try to identify and draw attention to the challenges in conducting CBD research for autism, offering guidance to overcome these barriers and promote consistency in reaching a definitive conclusion about the use of CBD in the vulnerable ASD population. This review aims to add an updated and comprehensive understanding of the current state of knowledge on this evolving topic.

## 2. Methods

### 2.1. Searching of Data

**Figure 1:**
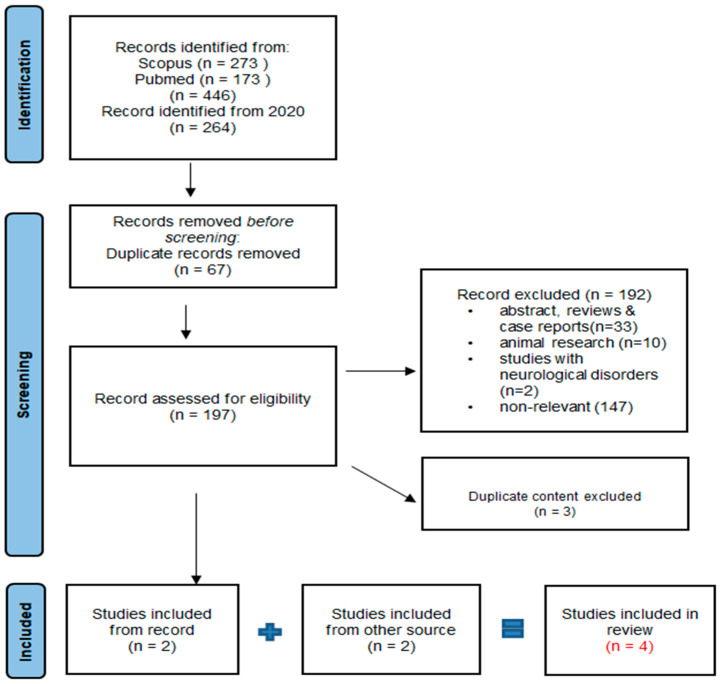
Study selection according to Preferred Reporting Items for Systematic Reviews and Meta Analysis (PRISMA) for evolving role of cannabidiol rich cannabis in people with autism spectrum disorder.

In February 2024, we performed a systematic evaluation after a thorough literature review under the guidelines of the Preferred Reporting Items for Systematic Reviews and Meta Analyses (PRISMA) [43]. The search was carried out in two databases: PUBMED and Scopus. The search strategy for the databases was formulated using terms identified in the title or abstract, encompassing keywords associated with cannabis like cannabidiol, cannabinoids, and CBD as well as those related to autism, such as autism spectrum disorder (ASD). We used the logical operators “AND” and “OR” for terms like “autism” and “cannabinoids” to search for studies in the databases. Our focus was on reviewing the potential of CBD as a possible treatment for alleviating symptoms associated with ASD. The focus of our review was to concentrate on studies specifically related to cannabidiol in individuals diagnosed with ASD with the goal of extending our understanding of the topic (Figure 1).

### 2.2. Selection Criteria

Initially, we included articles published up until February 2024 in any language and which involved human populations with a diagnosis of autism. We formed a duration filter of years and included all published research articles from 2020 onward until the date of our systematic review to highlight the most updated knowledge on our topic. We encompassed study populations of all ages and genders, and they were subjected to CBD use. We excluded all animal studies, case reports, abstracts, and review articles unrelated to the topic and studies which explained cannabinoid use for autism-like signs and symptoms of other neurological disorders.

### 2.3. Research Screening and Data Extraction

The initial screening involved reviewing the titles and abstracts to assess relevance, and only the articles deemed relevant to the topic were thoroughly reviewed in full, as per the inclusion criteria. Additional relevant studies were identified through the screening of references. Screenings of articles were initially performed by an independent author and then reviewed independently by another author. All disagreements were resolved by discussion, and the final selection of articles was made for this review. Every final selected article underwent extraction of data. All data were collected in a uniform information sheet. The scientific data and outcomes of an article extracted by an author were reviewed by another independent author in terms of their accuracy in extraction to decrease the risk of bias. Due to heterogeneity among the studies, we employed a qualitative synthesis to compare and summarize the data from the included studies. The extracted data were summarized descriptively, highlighting key patterns and variations among observations across different studies.

## 3. Results and Discussion

After the initial results, we found 446 articles. We refined our search by applying a filter for articles published from the year 2020 onward, resulting in 264 articles. Among these, 67 duplicates were removed. Additionally, 192 articles were excluded based on the predefined inclusion criteria. The reasons for exclusion included incorrect publication types, such as review articles, abstracts, or case reports (33), studies focusing on other neurological symptoms associated with ASD (2), animal research (10), and articles not relevant to the topic of cannabidiol use in autism (147). Subsequently, three more articles with duplicate content were eliminated. Ultimately, we identified two articles which met our criteria and were considered for inclusion in this review. By incorporating two additional articles sourced from Google Scholar, we expanded our review to encompass a total of four published articles for this qualitative synthesis (Figure 1).

### 3.1. Study Layout

This study included 353 ASD diagnosed participants in total within the age range of 5–25 years. The participants were both children and adults with no gender selections. Two studies from Israel and one each from Turkey and Brazil were included. The clinical studies consisted of three prospective and one retrospective studies [44], among which one study was open-label [45] while two were randomized, double-blind, placebo-controlled studies [46,47]. The period of study was in the range of 12–26 weeks.

Two studies had inclusion criteria for patients with ASD and behavioral problems [45,46]. One study reported 21 refusals from participation in the study prior to the treatment, with one of the reasons being behavioral problems [44]. Two studies had some participants who were under anti-epileptic treatment or anti-psychotic treatment [44,47]. The exclusion criteria of two studies were a long-term history of psychotic disorders [44,46], with a diagnosis of psychoses, schizophrenia or a schizoaffective disorder [45], and severe medical conditions which could possibly interfere with the study [47]. In all studies, participants who were on regular medications were advised to maintain their current regimens without altering them throughout the duration of the study.

### 3.2. Dosage of CBD-Enriched Cannabis

All studies used naturally derived medicinal cannabis products in oral dosage form with different concentrations. One study comparatively used cannabis whole plant extract and pure cannabinoids at concentrations of 167 mg/mL CBD and 8.35 mg/mL THC (6.7 mg CBD and 0.332 mg THC per drop) in drop form, respectively [46]. Others used CBD-enriched cannabis in sublingual drops at CBD concentrations of 500 mg, 1000 mg, and 2500 mg/ 30 mL with added trace THC [44], and one study used whole plant extract in drops which contained 5.7 mg CBD and 0.3 mg THC per drop [45]. Another used CBD-rich cannabis extract at a concentration of 5 mg/mL (0.5%) [47]. The medicinal cannabis extract or pure cannabinoids used had a high CBD:THC ratio of 20:1 in two studies [45,46] and 9:1 in one selected study [47], while another study used CBD preparation with full-spectrum CBD and a maximum of 0.3% trace THC [44].

We observed variations in the dosages administered in the studies. Initially, the dosage was selected according to each patient’s individual response, and adjustments were made as needed to ensure tolerability and efficacy. In one selected study, dosages ranged from 0.1 to 10 mg/kg of CBD and from 0.05 to 0.5 mg/kg of THC, with maximum doses of 420 mg of CBD and 21 mg of THC in a day [46]. Another study reported average dosages of 10 mg/kg of CBD and 0.5 mg/kg of THC, with the maximum doses not exceeding 400 mg of CBD or 20 mg of THC in a day [45]. A selected study reported an average dosage of 0.7 mg/kg, ranging from 0.3 to 2 mg/kg of CBD, with a maximum of 40 mg of CBD in a day [44]. One study used 3–70 drops per day [47].

### 3.3. Measuring Parameters

Among the included studies, all reported autistic participants benefit from medicinal cannabis, with improvements in their symptoms after treatment, but the measured parameters for analyzing the treatment outcomes varied. One study utilized standard measuring parameters like the Home Situations Questionnaire-Autism Spectrum Disorder (HSQ-ASD) and the Clinical Global Impression of Improvement (CGI-I) to target behavioral problems. The Social Responsiveness Scale, 2nd Edition (SRS-2) is a caregiver questionnaire which quantifies the severity of autism symptoms, while the Autism Parenting Stress Index (APSI) is a parent-rated measure which reflects a child’s behavior [46]. Two studies relied on clinical interviews with parents for assessment [44,47]. One study included in this review utilized standardized clinical behavioral assessments along with parent- and caretaker-based reports and the Autism Diagnostic Observation Schedule (ADOS) to assess the severity of ASD symptoms, with separate domains for social affect (SA) and restricted and repetitive behaviors (RRBs), administered by a well-trained speech therapist. The Social Responsiveness Scale (SRS-2) is a questionnaire completed by parents focusing on the behavior of a child, including social behavior. The Vineland Adaptive Behavior Scale (Vineland-3) involves a parent or caretaker interview conducted by a trained professional to assess adaptive functioning. Cognitive assessments are conducted using Wechsler Intelligence Scale subtests, which evaluate cognitive abilities relative to typical age groups [45].

### 3.4. Potential Improvements and Adverse Effects

Improvement was found in the symptoms of ASD participants in terms of behavioral abilities, social responses, and communication [44,46,47]. No significant improvement was seen in cognitive abilities in one study [45], while only four patients (12.9%) had improved cognition, as per Bilge and Ekici [44]. There was also a significant improvement in concentration in some mild ASD participants in another study [47]. Aran et al. did not directly assess this ability among their study participants. The targeted ASD symptoms were irritability, hyperactivity, anxiety, disturbances in sleep, and aggressiveness.

Somnolence, decreased appetite [46], restlessness [44], anxiety, increased aggression [45], dizziness, colic, and weight gain [47] were the primary adverse effects noted in our studies. One study reported a dropout rate of approximately 12%, with two patients experiencing seizures, three dropping out before treatment onset, two dropping out due to ineffectiveness, and others leaving due to side effects or other reasons [46]. In another study, one patient experienced a generalized seizure, and another showed increased stereotypical behaviors, leading to discontinuation of treatment due to these side effects [44]. Additionally, 28 participants from a separate study withdrew from the trial due to lack of improvement, noncompliance, and side effects [45] (Table 1). The substantial rate of discontinuation observed in the studies significantly influenced the overall conclusions.

### 3.5. Evaluation of CBD Efficacy and Challenges in ASD Treatment

ASD is a lifelong illness including a wide range of neurodevelopmental problems, characterized by difficulties with social interaction, communication, and the manifestation of repetitive or restricted behaviors [1]. There has been an increasing notion that medicinal cannabis can effectively reduce the behavioral symptoms which children and adults with ASD experience [36]. Determining how well therapy with cannabinoids works is challenging due to a lack of precisely quantifiable evidence on their safety, effectiveness, and mechanism of action [46,48]. In particular, the pathophysiology of inflammation, metabolic disorders, and neurotransmitter control is not currently characterized through available approaches.

There are already about 140 different phytocannabinoids known to exist in various versatile cannabis plants, along with a vast array of terpenoids, flavonoids, and other substances [49,50]. THC and CBD are more focused phytocannabinoids due to their influence on ECS, with THC being a plant psychoactive agent and CBD seeming to have anxiolytic, antiepileptic, antipsychotic, and neuroprotective qualities [51,52]. Since there is presently no FDA-approved treatment for ASD, the intention of this systematic review was to report whether CBD-based products benefited patients with ASD over the past few years and to consider their potential as a promising treatment option for the future.

Our analysis was based on four published studies which discussed the use of cannabidiol to help adults and children with ASD manage their symptoms. Variations in the study designs, sample sizes, evaluation criteria, length of therapy, and types of complications were found in these investigations. Furthermore, we found variations among the dosage and CBD source as well as the measurement methods which were adopted in evaluating participants’ progress. Following the initiation of CBD-rich cannabis treatment, it is noteworthy that all studies documented improvements in behavioral symptoms associated with ASD, but not all patients in the selected studies benefited from CBD treatment in equal proportions. The possible reasons behind this may be differences in the source or composition of the CBD products, differences in dosage per day for individual patients, or differences in age and gender among those selected for the study population. Certain genes are thought to have particular effects on cannabis. Different outcomes while using medical cannabis may be observed in certain people who have mutations in these receptors [53]. Studies have demonstrated that certain variations in the CNR1 gene, which codes for the CB1 receptor, can affect the effectiveness of cannabinoid therapy [54]. The heterogeneity of ASD makes it extremely challenging to suggest a suitable course of treatment, and patients display differences in the efficacy and safety of medicine [55].

In summarizing the findings more precisely, the evaluation of ASD symptom improvement relied on data provided by the parents or caregivers of the patients, yet there was inconsistency and no uniformity in the selection of outcome measures across studies. Also, according to recent reviews, there is variability in the outcome measures used in ASD research [35,36]. Researchers utilized various global assessment scales or clinical questionnaires without standardization. This underscores the necessity for confined and standardized assessment tools tailored to ASD symptoms to ensure consistency, particularly when assessing responses to drugs like CBD. Such tools should need to incorporate clinical scoring assessments alongside parental evaluations to mitigate potential biases and comprehensively address core ASD symptoms.

None of the studies conducted radiological or clinical blood tests to examine the effects of CBD on bodily functions, as all participants were receiving polypharmacy treatments, primarily antipsychotics and antiepileptic medications. The combined effects and interactions of these medications were not specifically addressed in recent research. There is a notable gap in the clinical trials for elucidating the pharmacological interactions between CBD and centrally acting drugs. Many centrally acting drugs are metabolized by cytochrome P450 iso-enzymes (CYP3A4, CYP2C19, CYP2D6, CYP2C9, and CYP1A2), which are inhibited by CBD [56,57]. Coadministration of these drugs may intensify adverse effects such as antimuscarinic syndrome, QT prolongation, and somnolence [58,59]. Future randomized controlled trials (RCTs) for ASD should focus on exploring these potential interactions, as they have the potential to alter clinical outcomes with synergistic, additive, or antagonistic effects.

Although no life-threatening effects were reported in the studies with CBD-rich cannabis, unlike those associated with major antipsychotics and other centrally acting drugs used for ASD symptoms, the observed severity of the side effects was typically mild to moderate, and they were often resolved with dose adjustments. This review underscores the importance of considering low-dose CBD to minimize the risk of adverse effects when administered alongside polypharmacy [60], as observed in our studies [44,47]. As a continuation of this, researchers found good safety outcomes for commercially available CBD-containing products administered at low daily doses in open-label [61] and double-blind, placebo-controlled clinical trials [62]. However, further research with larger sample sizes and reduced bias, such as in RCTs, is needed to provide more comprehensive insights into the ASD population. Common reported side effects include drowsiness, decreased appetite, weight loss, anxiety, euphoria, tiredness [46], restlessness [44], hyperactivity, increased aggression, weight gain, and intestinal colic [45,47], and some individuals reported a lack of improvement.

### 3.6. Regulatory and Ethical Obstacles in Conducting CBD Research for ASD

In recent years, there has been a scarcity of research regarding the medical uses of CBD for individuals with autism. Thus far, we have only conducted a few clinical trials in select parts of the world. The regulatory framework governing the medicinal use of CBD across various global locations serves as the primary constraint, thereby limiting the quantity, sample size, and quality of clinical trials conducted thus far [63,64]. The current legislative frameworks which govern the study of CBD exhibit notable variations among countries around the world, hence presenting obstacles to conducting rigorous clinical trials.

The research on cannabidiol and its effects on ASD describes a complex terrain. Previous reviews have focused on evidence from studies for the use of CBD in treating the symptoms of ASD. Our review also aimed to highlight and try to summarize the collective major challenges of conducting clinical trials on CBD in different jurisdictions of autism. However, navigating this research is challenging due to various factors. These include the unclear legal frameworks in some jurisdictions. In the European Union and other parts of the world, the legal frameworks for CBD products are ambiguous, which confuses consumers, preventing them from confidently using CBD products [65]. Different preparations containing CBD have been found to be contaminated and depend on the source origin of the cannabis. This poses a significant safety risk to consumers and requires rigorous testing and quality control measures [66]. Trust in using CBD products brings compliance in using CBD in vulnerable populations [67]. Similarly, there is a lack of standardization in the CBD market in different parts of the world due to varying potency and purity levels and the availability of limited data on the long-term effects of CBD use, which can make it difficult for researchers to ensure the safety and efficacy of the products they are using in their studies, particularly in populations like individuals with ASD. Additionally, the regulatory landscape for CBD is constantly evolving, with new regulations and guidelines being introduced, which can make it difficult for researchers to keep up to date and navigate the legal requirements. In some jurisdictions, CBD products are still considered illegal or subject to legal restrictions, which can limit the availability of CBD for research purposes and make it difficult to obtain the necessary permissions and approvals [68]. The FDA has not provided clear guidance on the use of CBD in human clinical trials, making it difficult for researchers to ensure that their studies are compliant with federal requirements [69,70]. When conducting research involving vulnerable populations like individuals with ASD, ethical considerations take priority, such as obtaining informed consent from the participants or their legal guardians and conducting thorough risk-benefit assessments to ensure that the potential benefits outweigh the risks [71]. The presence of these challenges highlights the need for concerted collaborative efforts which focus on addressing regulatory, scientific, and ethical factors in the field of CBD research for ASD. The shift from prescribed, controlled cannabis use to unregulated consumption poses significant risks for vulnerable individuals, being linked to neuropsychological deficits and other psychiatric issues. Comprehensive treatment strategies which integrate cognitive rehabilitation and ongoing support are essential to improve patient outcomes and reduce the risk of violence within this population [72]. Further research on CBD-rich cannabis is needed to clarify the causal relationships, and this requires clinical studies which create individualized treatment plans for these specific vulnerabilities.

### 3.7. Limitations and Future Directions

Furthermore, our systematic review was on topic-related published RCT studies as well as other clinical trials within an emerging field among researchers. This approach enabled us to offer the latest findings regarding the role of cannabidiol in ASD individuals.

The studies selected for our review exhibit several common limitations, such as small sample sizes, brief treatment durations, and variations in CBD product dosages and concentrations. Future clinical trials should address these limitations by implementing specific methodologies. These methodologies should aim to mitigate biases related to age-specific population groups, utilize standardized clinical assessment parameters, assess the impact of polypharmacy and comorbidity on clinical outcomes, evaluate CBD–drug interactions, explore dose–response relationships, and incorporate clinical investigations to assess bodily parameters. In this way, we can enhance our understanding of the efficacy and safety of CBD treatment for autism spectrum disorder.

We did not focus on ongoing clinical trials, and our review was on CBD among cannabinoids in ASD. Consequently, our database selection and scope were limited accordingly. Although the findings in our studies suggest promising results and a reduction in some ASD-related symptoms, we are unable to make conclusive comments on the role of CBD-rich cannabis in ASD. However, our review offers valuable insights that can provide guidance for future research and reviews, which might include new findings from ongoing registered or unregistered clinical studies.

## 4. Conclusions

Autism spectrum disorder presents a multifaceted challenge characterized by social, communicative, and behavioral difficulties. While there is growing evidence suggesting that CBD-rich cannabis could alleviate ASD symptoms, assessing its efficacy remains complex due to limited quantifiable evidence. We found social and behavioral improvement with CBD in our four selected studies with minimum adverse effects, which might be a promising alternative therapy for ASD in the future. Despite the positive outcomes observed in the studies, discrepancies in CBD product composition, dosage, and individual responses highlight the need for tailored treatment approaches. Standardized assessment tools are crucial for evaluating the efficacy of CBD consistently across multiple studies. Regulatory frameworks, ethical considerations, and challenges persist regarding the lack of clinical trials examining the interactions of CBD with other medications, necessitating further research for comprehensive insights into its safety and efficacy for ASD. Addressing these challenges requires collaborative efforts to standardize methodologies, expand sample sizes, and explore its long-term effects in ASD populations, which will ultimately enhance our understanding of its therapeutic potential.

## Figures and Tables

**Table 1:** Exploiting meta-analysis of different studies regarding methodology and findings related to behavior and cannabinoid administration.

Author	Study Methodology	Population Size	Age	Dose Details	Measuring Parameters	Findings	Summary
Aran et al., 2021 [46]	Randomized, double-blind, placebo-controlled crossover comparison of whole plant extract, pure cannabinoids, or placebo solution consisting of two 12 week treatment periods.	150 children and adolescents with ASD underwent treatment, with 12% attrition overall	5–21 years	During both treatment periods, the study participants received in drops either (1) whole plant extract CBD and THC at a ratio of 20:1 or (2) pure cannabinoids at a ratio of 20:1. The maximum daily intake was 420 mg of CBD and 21 mg of THC. Initial dosage: 0.05 mg/kg/d THC and 1 mg/kg/d CBD. Orally administered in three split doses, the daily total was titrated every other day based on body weight (20–40 kg: 0.5 mg/kg/d of THC and up to 10 mg/kg body weight of CBD per day; over 40 kg: 0.375 mg/kg/d of THC per day and 7.5 mg/kg body weight of CBD).	Baseline: ADOS-2, VABS, CARS2-ST Primary: HSQ-ASD, CG-I Secondary: SRS-2, APSI, adverse events	Participants who received whole-plant cannabinoids showed significant improvement in behavior (49%) (*n* = 45, *p* = 0.005) on the CGI-I compared with a placebo (21%) (*n* = 47), while a non-significant improvement (38%) was observed in the pure cannabinoids group (*n* = 45, *p* = 0.08). No significant differences were observed in the Home Situations Questionnaire-ASD (HSQ-ASD) or Autism Parenting Stress Index (APSI) scores between the groups. The Social Responsiveness Scale (SRS-2) significantly improved with whole plant extract (*n* = 34) compared with a placebo (*n* = 36) (14.9 vs. 3.6 points, respectively (*p* = 0.009)). No significant difference observed in those participants who received pure cannabinoids and a placebo (*p* = 0.801). Participants who received cannabinoids (whole-plant or pure) experienced a decrease in their baseline BMIs compared with a placebo. Common well-tolerated and mild adverse events included somnolence, decreased appetite, weight loss, tiredness, euphoria, and anxiety.	According to this study, cannabinoid treatment, particularly whole plant extract, may be able to benefit people with ASD by improving their primary symptoms and minimizing disruptive behaviors in a way that is tolerable.
Bilge & Ekici 2021 [44]	Retrospective analysis of CBD-enriched oral drops, with average treatment duration of 6.5 months in long observational study.	33 children with ASD (27 males and 6 females) underwent treatment, and 31 participants completed the study	Mean age in years = 7.7 ± 5.5	Two CBD-enriched cannabis brands were used with full-spectrum CBD and trace THC (less than 3%). Average daily dose of CBD-enriched cannabis was 0.7 mg/kg (0.3–2 mg/kg). The maximum dose of CBD was not higher than 40 mg/day.	Outcomes were assessed both before and after therapy based on clinical interviews and parental reports conducted during follow-up visits.	As per parents’ reports, the main improvements were a decrease in behavioral problems (*n* = 10, 32.2%), increase in expressive language (*n* = 7, 22.5%), improvement in cognition (*n* = 4, 12.9%), increase in social interaction (*n* = 3, 9.6%), and decrease in stereotypes (*n* = 1, 3.2%), with no change in daily life activity reported in 6 patients (19.35%). Adverse events reported were restlessness (*n* = 7, 22%), increased stereotypes (*n* = 1, 3%), and generalized seizures (*n* = 1, 3%).	This study on CBD-enriched cannabis treatment for autism spectrum disorder found that using lower doses of CBD and trace THC showed potential in treating behavioral issues related to autism without causing severe negative effects.
Hacohen et al., 2022 [45]	A prospective open-label study on whole plant extract drops for a period of 6 months.	110 participants with ASD (65 males) underwent treatment, and 82 participants completed the study	5–25 years	Cannabis whole plant extract in oil with CBD:THC ratio of 20:1. Initially one drop daily (drop contained 0.3 mg THC and 5.7 mg CBD) with gradual increase in dosage per response. Maximum dose of CBD was 10 mg/kg/day (or a total of 400 mg/day), and that for THC was 0.5 mg/kg/day (or a total of 20 mg/day).	Baseline and after 6 months: ADOS-2, age-appropriate Wechsler test, Vineland-3 scales, and SRS-2	Of those who initiated the study, 28 dropped out due to lack of cooperation (*n* = 8), lack of improvement (*n* = 8), or side effects (*n* = 12; increased aggression, anxiety, and hyperactivity, with some reporting weight gain and abdominal pain). Significant improvements were reported in the area of social communication; ADOS SA CSS showed significant improvement (*n* = 75, mean = −0.49, *p* = 0.001). Restricted repetitive behaviors: significant improvement in social scores (mean = −2.51, *p* = 0.038) and RRB scores (mean = −2.88, *p* = 0.014) on the SRS-2. Adaptive behaviors: significant improvement found on the Vineland-3 scale (*n* = 76, mean = 4.37, *p* < 0.001). Cognitive abilities: No significant changes, positive or negative, were found (*n* = 76). Inconsistency across measures was noted for changes in social communication skills.	This study indicates that treatment with CBD-rich cannabis in children and adolescents with ASD may lead to enhancements, especially in social communication skills, as observed through standardized clinical assessments.
Silva et al., 2022 [47]	Randomized, double-blind, placebo-controlled trial comparing treatment with CBD-rich cannabis extract and a control placebo for 12 weeks.	60 participants with ASD underwent treatment with CBD-rich cannabis extract (*n* = 31, male = 25) or a placebo (*n* = 29, male = 27)	5–11 years	Concentration of CBD-rich cannabis extract was 0.5% (5 mg/mL) with a CBD:THC ratio of 9:1. Three drops was the daily dose, gradually increasing by 2 drops twice in a week per day in line with improvement, with a maximum of up to 70 drops for the daily dose. The average number of drops being taken was 47.42 ± 15.22 (treatment group).	The outcomes were assessed using a semi-structured interview questionnaire from the caregivers both before and after the clinical trial.	Significant improvement found in CBD-rich cannabis extract treatment group (*n* = 31) for social interaction (*p* = 0.0002), anxiety (*p* = 0.016), psychomotor agitation (*p* = 0.0029), number of meals per day (*p* = 0.04), and concentration (significant in mild ASD cases; *p* = 0.01), while 3 children (9.7%) had mild adverse effects like colic, weight gain, dizziness, and insomnia.	Study findings suggest that CBD-rich cannabis extract might be a promising therapeutic for children with ASD, being found to significantly improve social interaction and emphasizing the need for further research to confirm these results and determine the optimal dosage and treatment duration.

## Abbreviations

ASD	Autism spectrum disorder
FDA	Food and Drug Administration
ECS	Endocannabinoid system
eCBs	Endocannabinoids
AEA	Anandamide
2-AG	2-arachidonoylglycerol
THC	Δ9-tetrahydrocannabinol
CBD	Cannabidiol
CB	Cannabinoid receptor
RCTs	Randomized controlled trials
FAAH	Fatty acid amide hydrolase
TRPV	Transient receptor potential cation channel subfamily V member
GABA	γ aminobutyric acid
GPR55	G protein coupled receptor 55
PPARγ	Peroxisome proliferator activated receptor gamma
5-HT	Serotonin 5HT receptor
